# 585 Focused Wound Care Handoff Improves Burn Center Physician-Nursing Communication and Wound Care Education

**DOI:** 10.1093/jbcr/irac012.213

**Published:** 2022-03-23

**Authors:** Sohayla Rostami, Qingwen Kawaji, Stephanie L Martinez, Tomer Lagziel, Rowena Orosco, Carolina J Flores, Charles S Hultman, Julie Caffrey

**Affiliations:** Johns Hopkins University, Baltimore, Maryland; Johns Hopkins Hospital, Baltimore, Maryland; Johns Hopkins, Towson, Maryland; Johns Hopkins University School of Medicine / Sackler School of Medicine, New-York Program, Tel-Aviv University, Rockville, Maryland; Johns Hopkins Bayview Medical Center, Baltimore, Maryland; Johns Hopkins Bayview Medical Center, Rosedale, Maryland; Johns Hopkins University School of Medicine, Baltimore, Maryland; Johns Hopkins, Baltimore, Maryland

## Abstract

**Introduction:**

Burn patients often require changing wound care routines dependent on wound characteristic and operative interventions. Unfortunately, order discrepancies on electronic medical systems, poor communication between providers, nursing, and wound care technicians leads to incorrect wound care treatment. By creating a daily dedicated wound care discussion involving integral components of the wound care team (provider, charge nurse, and wound care technicians), we hope to improve communication amongst team members and provide wound care education at all levels.

**Methods:**

The study was carried out at a single-center burn unit. A pre-intervention, de-identified survey was distributed to the nursing staff to determine familiarity with wound care as well as assessment of communication regarding wound care in the burn unit. A planned intervention was then initiated for a period of four weeks. Daily, timed dedicated wound care rounds were carried out. The wound plan was then reflected on a personalized diagram for each patient, on electronic medical records and updated on resident/fellow notes. Nursing staff satisfaction and assessment of communication was completed again using a post-intervention, de-identified survey.

**Results:**

Initial data from our planned four-week intervention showed that on average, we round on 7.9 patients on burn wound unit, 4 patients on intermediate care unit (IMC), and 2.9 patients on intensive care unit daily. During wound care handoff, we discussed 12.5 patients daily with an average time of 51 seconds per patient. When wound care routines are added to morning rounds, we spend 50 seconds per each burn wound unit patient, 120.5 seconds per each IMC patient and 735 seconds per each ICU patient. There was a total of 19 surveys collected prior to intervention. Four of the 19 surveys were filled by burn wound floor nurses and 15 were filled by ICU nurses. On average, nursing reported their wound care proficiency to be 4 out of 5 (5 being the highest score). Majority reported spending about 1-3 hours per shift on wound care (58%). Most nurses review wound care routine on nursing handoff (66%), resident/fellow notes (22%) and electronic medical record orders (11%). Mid-intervention satisfaction survey showed that most nurses felt that communication was improved, discrepancies between staff members were minimized, and that 100% of nurses wanted this intervention to be continued.

**Conclusions:**

Adverse wound care events can happen despite nursing self-reported high level of wound care proficiency. To ensure accurate plan of care, wound care routine needs to be communicated at multiple levels: during rounds, in daily progress notes, and on electronic medical record orders.

